# Changing Patterns of Malaria Epidemiology between 2002 and 2010 in Western Kenya: The Fall and Rise of Malaria

**DOI:** 10.1371/journal.pone.0020318

**Published:** 2011-05-23

**Authors:** Guofa Zhou, Yaw A. Afrane, Anne M. Vardo-Zalik, Harrysone Atieli, Daibin Zhong, Peter Wamae, Yousif E. Himeidan, Noboru Minakawa, Andrew K. Githeko, Guiyun Yan

**Affiliations:** 1 Program in Public Health, College of Health Sciences, University of California Irvine, Irvine, California, United States of America; 2 Climate and Human Health Research Unit, Kenya Medical Research Institute, Kisumu, Kenya; 3 Department of Medical Entomology, Institute of Tropical Medicine, Nagasaki University, Nagasaki, Japan; Menzies School of Health Research, Australia

## Abstract

**Background:**

The impact of insecticide treated nets (ITNs) on reducing malaria incidence is shown mainly through data collection from health facilities. Routine evaluation of long-term epidemiological and entomological dynamics is currently unavailable. In Kenya, new policies supporting the provision of free ITNs were implemented nationwide in June 2006. To evaluate the impacts of ITNs on malaria transmission, we conducted monthly surveys in three sentinel sites with different transmission intensities in western Kenya from 2002 to 2010.

**Methods and Findings:**

Longitudinal samplings of malaria parasite prevalence in asymptomatic school children and vector abundance in randomly selected houses were undertaken monthly from February 2002. ITN ownership and usage surveys were conducted annually from 2004 to 2010. Asymptomatic malaria parasite prevalence and vector abundances gradually decreased in all three sites from 2002 to 2006, and parasite prevalence reached its lowest level from late 2006 to early 2007. The abundance of the major malaria vectors, *Anopheles funestus* and *An*. *gambiae*, increased about 5–10 folds in all study sites after 2007. However, the resurgence of vectors was highly variable between sites and species. By 2010, asymptomatic parasite prevalence in Kombewa had resurged to levels recorded in 2004/2005, but the resurgence was smaller in magnitude in the other sites. Household ITN ownership was at 50–70% in 2009, but the functional and effective bed net coverage in the population was estimated at 40.3%, 49.4% and 28.2% in 2010 in Iguhu, Kombewa, and Marani, respectively.

**Conclusion:**

The resurgence in parasite prevalence and malaria vectors has been observed in two out of three sentinel sites in western Kenya despite a high ownership of ITNs. The likely factors contributing to malaria resurgence include reduced efficacy of ITNs, insecticide resistance in mosquitoes and lack of proper use of ITNs. These factors should be targeted to avoid further resurgence of malaria transmission.

## Introduction

Malaria is the leading cause of child mortality and morbidity in Africa. There is growing evidence documenting a substantial decline in clinical malaria morbidity and mortality in a number of African countries, including Kenya. This can be attributed to malaria control measures, predominantly to the use of insecticide treated nets (ITNs), indoor residual spray (IRS) and artemisinin-based combination therapies (ACT), which have been implemented on a wide scale [1]–[6]. Encouraged by the progress made in the last decade, malaria elimination and eradication has been set as the new goal of the World Health Organization malaria strategy [7], [8]. However, there are also attribution challenges regarding the reported progress [9]–[11].

ITN coverage and reported malaria incidence are not spatially congruent, and remarkable success has been reported in reducing malaria prevalence and mortality in different settings in Kenya [12]–[17], but the mechanism behind the declining malaria burden is debatable [14], [18]–[23]. Some studies [5], [22], [23] found that malaria reduction was associated with the increased use of ITN coverage and IRS, while other studies [13], [14] demonstrated that the change from a failing drug (chloroquine) to a more effective drug (sulphadoxine plus pyrimethamine or an artemisinin combination) led to immediate improvements. Additional studies [15], [17], [18] also found that a decline in malaria incidence began several years before the scale-up of malaria control and the availability of ACT drugs. Furthermore, recent studies in Kenya reported elevated malaria trends in a number of health facilities [17], [18]. So far, the changing trend in malaria incidence has been shown mainly through data or surveys from health facilities, where most records are based on presumptive treatment with only a small portion based on laboratory confirmed diagnoses. Thus, they are not reliable estimates of the true status of malaria transmission in the general population because any improvement in diagnosis and treatment will appear to make a great difference [24], [25].

ITNs have been used in rural Kenya since the early 1980s, but the coverage has been low. In 2002, the Ministry of Health (MoH) of Kenya developed an intensive ITN delivery programme targeting pregnant women and children under the age of 5 years. The goal was to achieve a rapid scale-up in net coverage and to reach a target of 60% coverage of populations at risk by 2005 through the commercial retail sector. However, due to persistent low net coverage [20], a new policy intervention initiated in 2004, resulted in the distribution of heavily subsidized nets through health facilities in 2005. From June to September 2006, through the support of the Global Fund, the Kenya MoH implemented a mass community-based distribution of long-lasting insecticide-impregnated bed nets (LLINs) and distributed 7.1 million ITNs free of charge, of which 6.3 million were LLINs [1]. These new intervention policies may have greatly impacted vector abundance and malaria parasite prevalence. However, all earlier studies lack critical long-term monitoring and evaluation of the epidemiological and entomological impacts of the new malaria control measures. Moreover, World Health Organization has issued guidelines on the proper use and sustainability of high coverage of bed nets in 2005 [26], which include purchase/distribution of only LLINs, replacement of old LLINs (3–5 y), among others. There was reported misuse of ITNs, but assessment on the efficacy of ITN/LLINs in real-time and replacement of aged ITNs/LLINs is lacking, which may affect the sustainability of effective coverage and thus, the continuity of suppression on vector population and parasite transmission.

Here, we report on a nine-year longitudinal survey of both monthly malaria parasite prevalence in school children and vector abundances in one endemic mid-altitude site and two epidemic highland sites in western Kenya, to assess the impact of ITN/LLIN coverage on malaria parasite transmission and the malaria vector population. The limitations of using ITNs as a primary prevention measure to combat malaria are also discussed.

## Methods

### Study areas

The study was conducted in three sentinel sites in western Kenya: two highland sites, one in Iguhu (34°45′E, 00°10′N, 1,430–1,580 m a.s.l) (mesoendemic) in the Kakamega district and one in Marani (34°48′E, 00°35′S, 1,540–1,740 m a.s.l) (hypoendemic) in the Kisii district, and one mid-altitude site in Kombewa (34°30′E, 00°07′N, 1,150–1,300 m a.s.l) (holoendemic) in the Kisumu district. The catchment populations range from 19,000 in Marani, to 23,000 in Kombewa and 24,000 in Iguhu. The topography of the two highland sites is characterized by valleys and depressions surrounded by densely populated hills. The mid-altitude site has a rolling terrain bisected by small streams. The climate in western Kenya generally consists of a bimodal pattern of rainfall, with the long rainy season from April to June, which triggers the peak malaria transmission period, and the short rainy season from October through November. The dry season in Kenya, when the overall rainfall is significantly less than in the wet seasons, occurs from July to September and it is also the coolest season. January and February are the hottest months. *Plasmodium falciparum* is the primary malaria parasite species [27], and the predominant malaria vector species are *Anopheles gambiae* s.s., *An*. *arabiensis* and *An*. *funestus* [28], [29].

### Parasitological survey

Monthly parasitological surveys were initiated in June 2002, and in February and June 2003 in Iguhu, Marani, and Kombewa, respectively. The parasite surveys were conducted using randomly selected school children aged 6–13 years in all study sites. At least 100 volunteer school children from 5–6 primary schools were sampled at each site and each month for parasite prevalence determination since 2002. During high-transmission seasons (May and June each year), we increased the sample size to up to 300 volunteers to increase the statistical power for parasite prevalence determination. The sample size was calculated based on the size of study population and parasite prevalence from the previous study [27]. Finger-prick blood samples were collected, and thick and thin smears were prepared for microscopic species identification and parasite counts. Parasite species and gametocytes were identified microscopically. Malaria parasite counts were read against 200 white blood cells, and density was expressed as parasites per μl assuming a count of 8,000 white blood cells per μl. The detailed procedure for quality control of blood smear reading was described previously [27].

### Entomological survey

Longitudinal mosquito adult surveys were started in February 2002, and in February and June 2003 in Iguhu, Marani, and Kombewa, respectively. In each site, 30–40 houses were randomly selected for alternate monthly adult mosquito sampling to eliminate any post spray effect. Indoor resting female mosquitoes were collected using the pyrethrum spray catch (PSC) method [30]. The number of sleepers in each house was recorded during the surveys. Vector species were morphologically identified as either *An. funestu*s or *An. gambiae* s.l. [31].

Adults of the *An. gambiae* complex were further analyzed by three PCR methods. Mosquitoes collected from 2002–2005 were analyzed by the PCR method described in a previous study [29]. From 2006 onward, only legs were used for PCR species identification. About one-third of the leg samples were analyzed by DNA extraction and amplification of the ribosomal DNA using the Terra-Direct PCR method (Clonetech, Mountain View, CA. The rest of the samples from 2006 to 2010 were analyzed by using Fast Tissue-to-PCR kit (Fermentas, Glen Burnie, MA) and individual specimen was identified by the standard PCR procedure [29].

### Surveys of insecticide treated nets

Cross-sectional ITN coverage surveys on randomly selected households were conducted from late 2003 to early 2004, and from January 2006 through October 2010 in the study sites. Information obtained from a household questionnaire survey included number of bed nets owned, number and ages of persons sleeping under bed net, age and condition of bed net (torn or not), types of bed net (regular ITN or LLIN), and retreatment of regular ITNs (retreated every 6 months, more than 6 months, or not at all). The number of LLINs owned was recorded in surveys from 2008 onwards.

Ownership of ITNs did not necessarily directly translate to regular usage by either children or adults. An additional questionnaire on actual usage of bed nets was carried out during surveys in early 2009 and 2010 focusing mainly on who (sex and age) and how often (everyday, used it but not everyday, or not used at all) the residents of the household used the ITNs.

### Contact bioassay of bed net insecticidal efficacy test protocol

Bed nets of different ages were randomly collected in 2009 and 2010 from households that owned bed nets. We replaced these nets with the new ITNs being distributed by the Kenya MoH through government hospitals. Thirty nets were tested at each site.

The insecticidal efficacy of nets was evaluated by a slightly modified WHO [32] bioassay procedure using a paper cup, instead of a plastic cone, for a 3 minutes exposure of female *An. gambiae* s.s. (susceptible Kisumu strain). The Kisumu strain of *An. gambiae* s.s. is a standard susceptible reference strain originally colonized in western Kenya used in previous studies [33]. Each bed net sample was tested by exposing about 25 female mosquitoes, and three samples (from different sides of the net) per net were tested. Mosquitoes were maintained on a ball of cotton wool soaked in a 10% sugar solution and allowed to recover for 24 hours. Mosquito mortality was scored 24 hours post exposure. Mortality rate in the control was <5% in every case, with no knockdown within 3 min exposure to the untreated net. The tests were carried out in June 2009 and September 2010.

### Temperature and precipitation data

Mean monthly weather records from 2002 to 2010 were obtained from the meteorological stations in Kakamega (for Iguhu), Kisii (for Marani) and Kisumu (for Kombewa). These meteorological stations were within 20 km from the study sites. The parameters used in this study include monthly average of daily maximum, mean, and minimum temperature, and monthly cumulative rainfall.

### Scientific and Ethical clearance

Scientific and ethical clearance was given by the Kenya Medical Research Institute and University of California at Irvine institutional review boards. Volunteers were enrolled from the primary schools in the study sites through the primary school administrators with the permission of the division office of the Ministry of Health. Written assent for children (<18 years of age) were obtained by the participants and their parents or guardians. Inclusion criteria included: provision of informed consent, age >6 months at recruit, and no reported chronic or acute illness except malaria. Exclusion criteria include: those who were unwilling to participate in the study, and those who planned to move out of the study area. According to the standard malaria treatment guidelines of the Ministry of Health of Kenya, asymptomatic infections were not treated with antimalarials, but symptomatic volunteers were referred to the local government hospitals or clinics for diagnosis and treatment free of charge.

### Statistical analysis

*Plasmodium* parasite prevalence was expressed as the ratio of positive samples over the total number of samples tested. The abundances of anopheline mosquitoes in each site were computed monthly as the number of females per house per night (f/h/n). Bed net ownership rate was calculated as the ratio of the number of households with at least one bed net over the total number of households surveyed. Population coverage with bed nets was computed as the ratio of the total number of individuals reporting sleeping under a bed net, regardless of the condition and type of the net, over the total number of individuals surveyed. Effective population coverage by bed net is defined as the proportion of the population who slept under LLINs in good conditions, or ITNs in good conditions and routinely being re-treated by insecticides. Ineffective population coverage by bed net is defined as the proportion of the population who slept under torn nets, or slept under regular ITNs but those that were not routinely retreated by insecticides [34], [35]. The differences in vector abundances and parasite prevalence between different time periods at each of the three surveyed sites were compared using the Tukey-Kramer HSD test of ANOVA with repeated measures. The differences in efficacy among different age groups of ITN/LLINs were compared using the Tukey-Kramer HSD test of ANOVA. Changes in vector species composition over time were compared using the χ^2^-test. Bed net ownership was compared between different survey periods using the χ^2^-test.

To find the trends in the parasite prevalence or vector density dynamics, we performed time-series analyses in the following three steps. First, due to seasonal fluctuations in prevalence and vector density, we used the 12-month moving average to minimize the effect of seasonality. Second, we examined the existence of time-varying trend using the randomness and stationarity tests of differenced series. The randomness of a series was tested using autocorrelation function (ACF) and partial correlation function (PCF), and stationarity was tested by examining whether the mean and variance were constant in time [36]. If a series is stationary and random with the first-order differencing, then the series has a constant trend; a series has a time-varying trend if it is stationary and random with second-order differencing. Third, if the 12-month smoothed data exhibit a trend, we fit the 12-month smoothed parasite prevalence or vector density time series data to appropriate trend models using the ordinary least square (OLS) method [36]. Two models were selected. For parasite prevalence data from Kombewa, the following nonlinear model was selected,
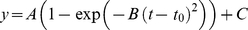
(1)where *y* is parasite prevalence, *t* is time, *A*, *B*, *C* and *t*_0_ are parameters. The lowest prevalence month, *t*_0_, was in July 2007.

For the 12-month smoothed parasite prevalence data in Iguhu and Marani and the 12-month smoothed vector density series in all three study sites, the following model was used:
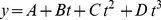
(2)where *t* is time, *A*, *B*, *C* and *D* are parameters. All parameters, except *t*_0_, were estimated using the least square method of nonlinear estimation.

For association between climatic factors and vector density and parasite prevalence, time-lagged cross-correlations were computed based on monthly observations, and a maximum of 3-month time-lag has been used. Software package STATISTICA 8.0 (StatSoft, Tulsa, USA) was used to perform all analyses.

## Results

### Descriptive parasite and vector data

Over the nine year period, we read a total of 33,640 blood smears, and malaria parasite prevalence rates were 47.1% (2,959/6,462), 28.4% (5,643/20,239), and 6.2% (351/5,639) in Kombewa, Iguhu, and Marani, respectively. *Plasmodium falciparum* was the predominant parasite species, constituting 97.3% to 98.3% of total positive blood samples, and *P. malariae* comprised 2.3–5.1% and *P. ovale* less than 1%. There were a few cases of mixed infections of two parasite species in all study sites but the rates of mixed infections were generally low. Due to the low prevalence of other parasite species, only *P. falciparum* prevalence dynamics was analyzed in the following sections.

Malaria vectors in the three sites comprised *An. gambiae* s.l. and *An. funestus*, with *An. gambiae* s.l. accounting for 37.4%, 86.9%, and 67.8% in Kombewa, Iguhu, and Marani, respectively. Thus, *An. funestus* was the predominant malaria vector in Kombewa, the mid-altitude area, but *An*. *gambiae* s.l. dominated in the highlands.

### Changes in *Plasmodium* parasite population dynamics

**Figure 1:**
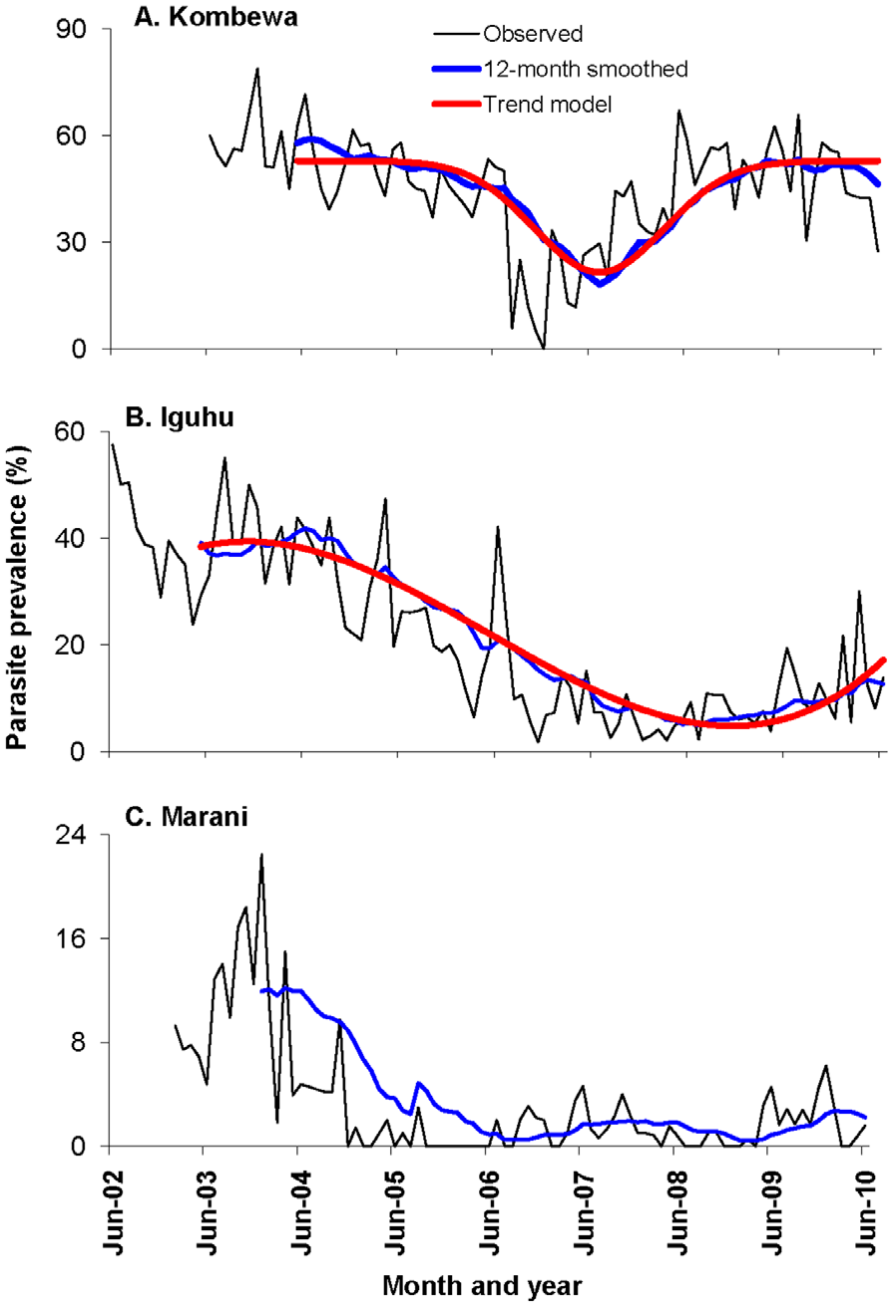
Dynamics of *Plasmodium falciparum* parasite prevalence (%) from 2002 to 2010. Study site from top to bottom: Kombewa, Iguhu, and Marani. The timings of major changes in ITN/LLIN distribution policy were marked on top.

Similar trends were observed in all three sites, i.e., declining parasite prevalence from 2002/2003 to 2006/2007, and a rebounding trend in prevalence from 2007 to present, with current parasite prevalence comparable to that five years ago (Fig. 1).

In Kombewa, parasite prevalence decreased slightly from 2003 to June 2006 (average 52.8%, range 37–78%), then declined sharply (average 8.9%, range 0–25%) during the last half of 2006 (Tukey-Kramer HSD test, P<0.0001). Thereafter it gradually increased throughout 2007 (average 30.0% in 2007) and well into 2008, reaching a monthly rate of 49.6% in 2008, about the same prevalence observed before 2006 and significantly higher than 2007 (Tukey-Kramer HSD test, P<0.05) (Fig. 1). Parasite prevalence remained consistently high since 2008.

In Iguhu, a decreasing trend in *P. falciparum* prevalence was observed from 2002 with a notable decline in 2005; however, a sharp declining trend occurred after June 2006 (Fig. 1). The monthly parasite prevalence dropped from an average of 33.8% (range from 18–57%) before July 2006 to 7.5% (range from 2–16%) between July 2006 and December 2008 (Tukey-Kramer HSD test of ANOVA with repeated measure, P<0.0001). Monthly mean parasite prevalence was 13.0% in 2009, which significantly exceeded the 2007/2008 level. The first six months of 2010 recorded a 16.9% average prevalence, which was two times higher than the mean of the same periods from 2007 to 2009 and was similar to parasite prevalence five years ago.

The temporal variations in parasite prevalence in Marani were very similar to those in Iguhu. In Marani, the decreasing trend in parasite prevalence started from early 2004 when the prevalence was down from 10.1% before 2005, to 1.1% from 2005 to 2008 (Fig. 1, Tukey-Kramer HSD test, P<0.001). Mean monthly parasite prevalence was 2.0% from 2009 to 2010, which was slightly but not statistically higher than the average from 2005 to 2008.

**Figure 2:**
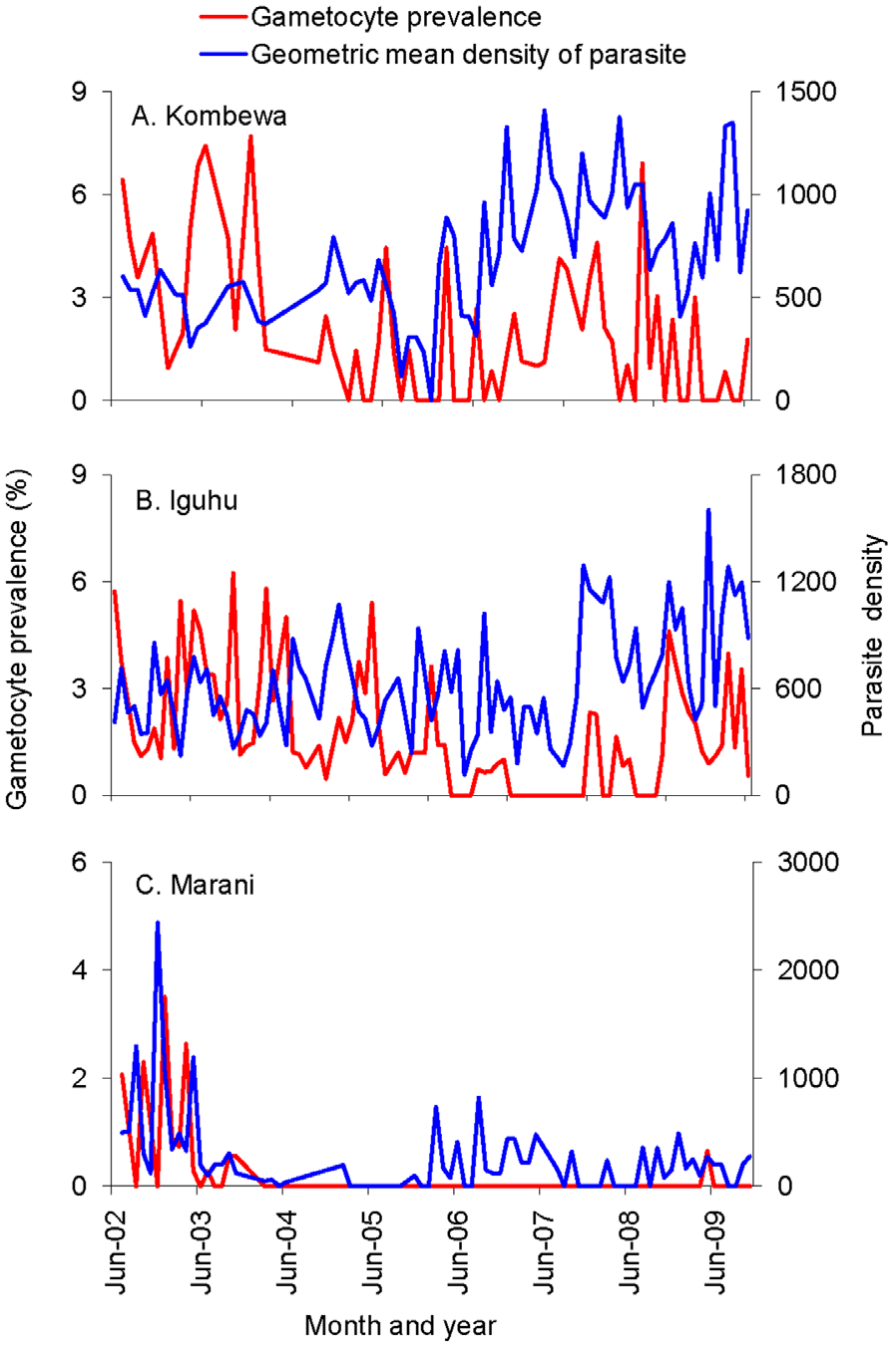
Dynamics of *Plasmodium falciparum* infection intensity and gametocyte prevalence (%) from 2002 to 2010. Study site from top to bottom: Kombewa, Iguhu, and Marani.

It is interesting to note that the mean malaria parasite density in infected volunteers exhibited an increasing trend from 2007 to 2009 and fluctuated in 2010 in Kombewa (Fig. 2A) and Iguhu (Fig. 2B). The increased parasite density was probably linked to waning immunity after the major scaling-up of ITNs in 2006. In Marani, parasite density remained low throughout the study period (Fig. 2C). There was no clear trend for gametocyte prevalence for all three sites (Fig. 2).

### Variation in vector densities and species composition

**Figure 3:**
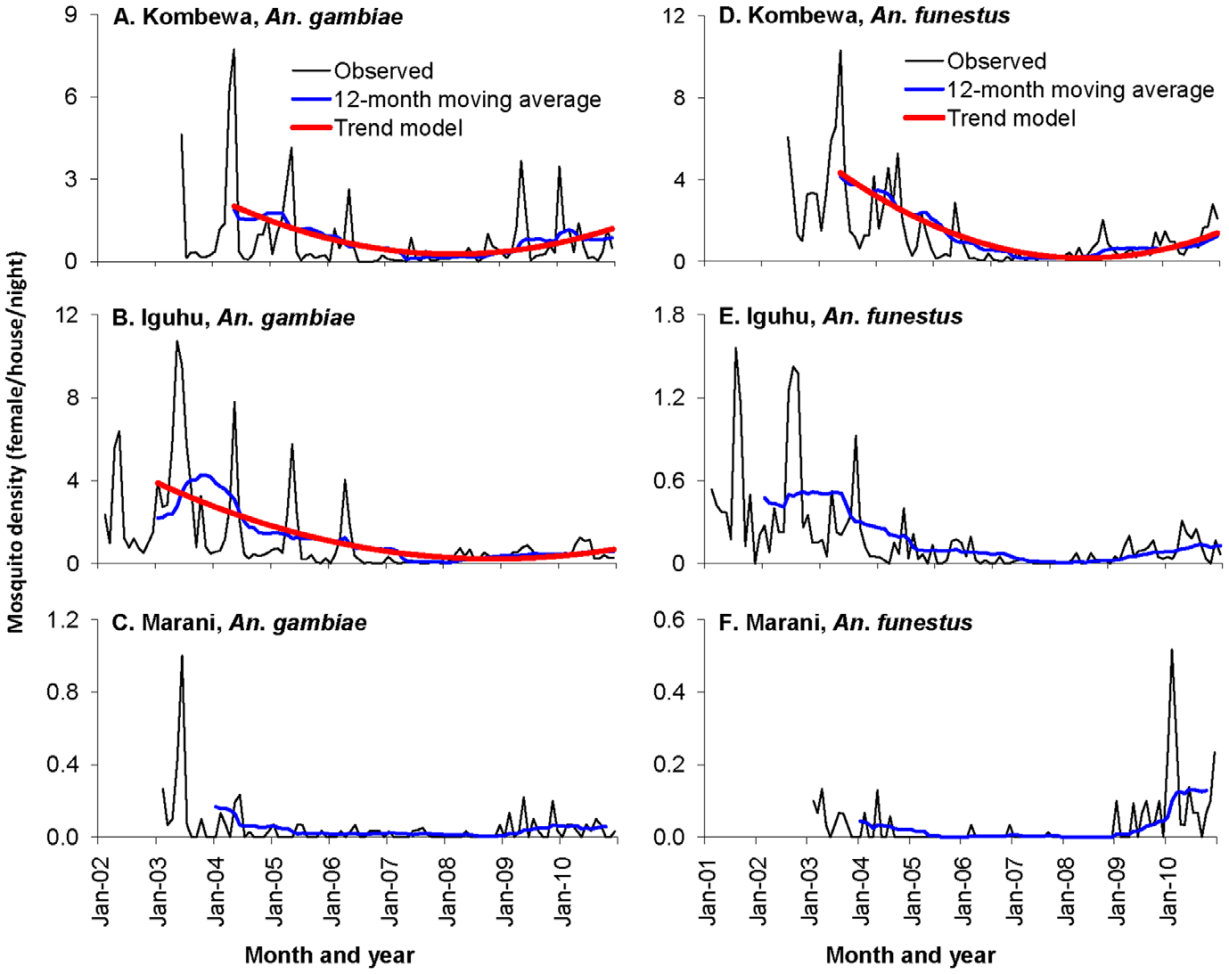
Temporal dynamics of *Anopheles gambiae* s.l. (left panel) and *An. funestus* (right panel) density (number of females/house/night) from 2002 to 2010.

The mean abundances of indoor resting *An. gambiae* s.l. and *An. funestus* varied significantly in all sites. In both Kombewa and Iguhu, mosquito abundances declined by 90% from 2002 to 2007 (Fig. 3), with *An. gambiae* s.l. abundances decreasing from 2.2 females/house/night (f/h/n) and 1.3 f/h/n to 0.24 f/h/n and 0.23 f/h/n after July 2006 in Iguhu and Kombewa, respectively (Tukey-Kramer HSD test, P<0.0001). Meanwhile, *An. funestus* densities declined from 0.3 f/h/n and 4.4 f/h/n to 0.03 and 0.4 f/h/n in Iguhu and Kombewa, respectively during the same period (Tukey-Kramer HSD test, P<0.0001). Similar to malaria parasite prevalence, malaria vector densities have also resurged since 2007. Compared with 2007, indoor resting abundances of *An. gambiae* s.l. and *An. funestus* increased by 8–10 folds in Iguhu in 2009 and 5–7 folds in Kombewa. The mean monthly density of *An. gambiae* s.l. from January to March 2010 was 1.8 f/h/n in Kombewa, which was the highest since 2003 and was significantly higher than the second highest density of 0.7 f/h/n in 2004. In Marani, the indoor resting vector densities were all below 0.5 f/h/n except in June 2003 when it was 1.0 f/h/n (Fig. 3). Malaria vector densities in Marani also showed some reduction from 2003 to 2006, but have rebounded since 2007.

**Figure 4:**
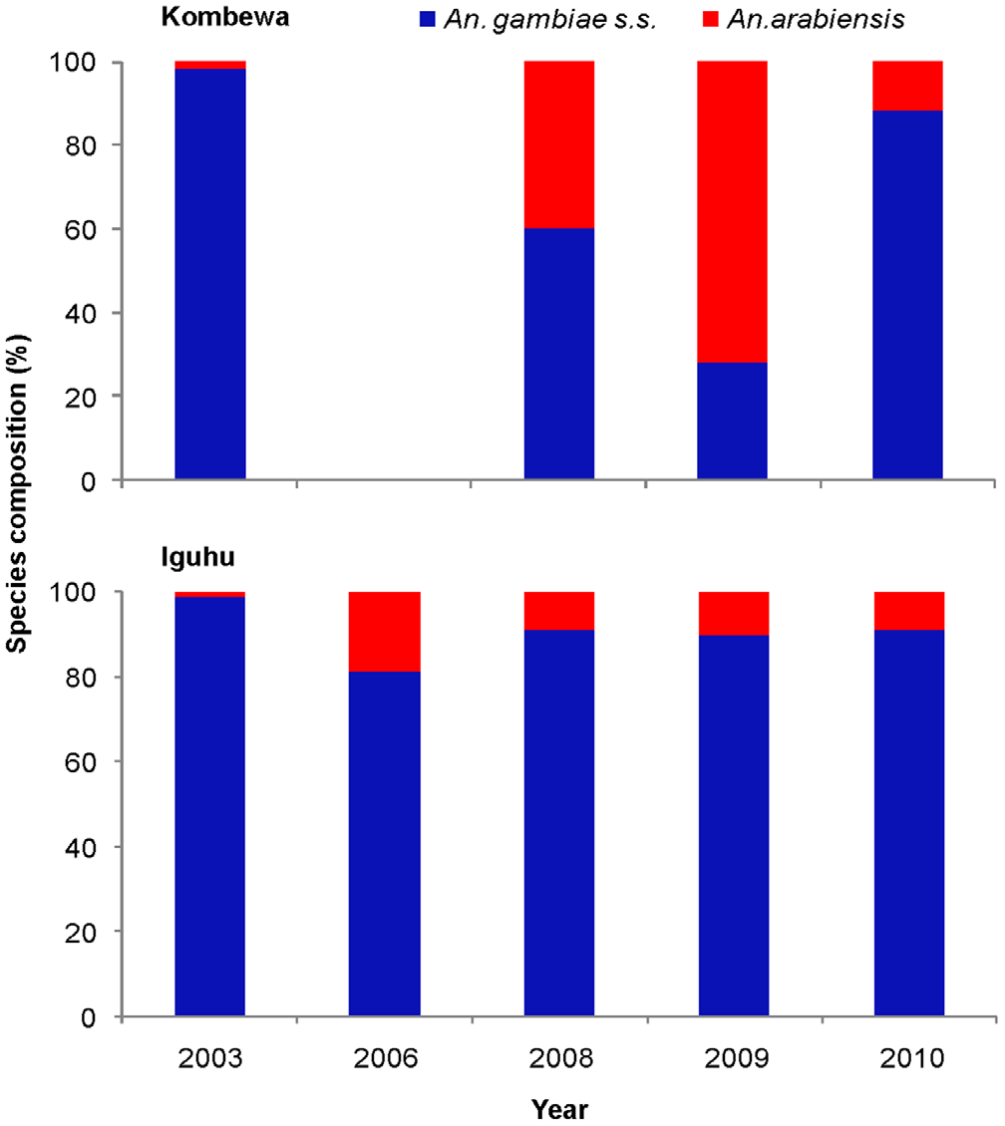
Species composition of *Anopheles gambiae* s.s. and *An. arabiensis* in Kombewa (top) and Iguhu (bottom) from 2003 to 2010.

Mosquito species composition reflects a combination of environmental and historical intervention events at a site; hence, changes in species composition can provide a sensitive measure of ecological changes. Previous studies show that use of ITNs may have a substantial impact on vector species composition, especially on the replacement of the anthropophilic and endophilic *An*. *gambiae* s.s. by the zoophilic and exophilic sibling species, *An*. *arabiensis*, in areas where ITN use has increased [37], [38]. Figure 4 shows the changes in species composition of the *An*. *gambiae* s.l. complex from 2003 to 2010 in Kombewa and Iguhu. Analysis of species composition illustrated that the proportion of *An*. *arabiensis* rose significantly in Kombewa from 1.7% in 2003 to 61.7% in 2009 (χ^2^ = 65.8, d.f.  = 1, P<0.0001), but decreased significantly to 11.5% in 2010 (compared with 2009, χ^2^ = 119.5, d.f.  = 1, P<0.0001). In contrast, species composition change in Iguhu was characterized by a significant increase in the proportion of *An*. *arabiensis* from <1% in 2003 to 18.8% in 2006 (Fisher exact test P<0.001), then by a gradual declining trend from 2006 to 2010 (9.2%, Fisher exact test P<0.05) (Fig. 4). Due to the small number of samples, *An*. *arabiensis* from Marani was not analyzed.

#### Trend analysis of parasite prevalence and vector density time series

The 12-month smoothed prevalence time series were shown in Figure 1. Prevalence series from Iguhu and Kombewa were not stationary after the first-order differencing, but were stationary and random after the second-order differencing. This indicates that these prevalence series have a time-varying trend after seasonal effect was canceled out. The prevalence series from Marani was not stationary after the first-order differencing, but it was over differenced after the second-order differencing because both ACF add PCF values were significantly negative after the second-order differencing, implying that parasite prevalence at this site was a random fluctuation over time with no time-varying trend. Goodness-of-fit result demonstrates that model (EQ 1) exhibited the best fitting for parasite prevalence data of Kombewa with an adjusted *R* ^2^ = 0.96 (Fig. 1 and Table 1), while model (EQ 2) fits best for parasite prevalence data of Iguhu with an adjusted *R* ^2^ = 0.98 (Fig. 1 and Table 1). Trend models showed that parasite prevalence has resurged since late 2007 in Kombewa and late 2008 in Iguhu.

**Table 1:** Parameter estimation of the trend model analysis. Models were only built for series where time-varying trend were detected.

Site	Parameter	Estimation (95% CI)	*t*-value	*p*-level
Kombewa	Parasite prevalence, *F*_3,71_ = 988.20, *P*<0.0001, adjusted *R* ^2^ = 0.96			
	A	31.349 [29.779, 32.920]	39.804	<0.0001
	B	0.008 [0.007, 0.008]	15.278	<0.0001
	C	21.522 [20.043, 23.001]	29.012	<0.0001
Iguhu	Parasite prevalence, *F*_3,82_ = 1662.10, *P*<0.0001, adjusted *R* ^2^ = 0.98			
	A	28.694 [25.191, 32.196]	16.298	<0.0001
	B	1.309 [1.065, 1.553]	10.679	<0.0001
	C	−4.527 [−5.019, −4.034]	−18.285	<0.0001
	D	0.315 [0.285, 0.345]	20.980	<0.0001
Kombewa	*Anopheles gambiae* density, *F*_2,77_ = 233.71, *P*<0.0001, adjusted *R* ^2^ = 0.85			
	A	3.059 [2.842, 3.277]	28.033	<0.0001
	B	−0.096 [−0.106, −0.087]	−20.322	<0.0001
	C	0.083 [0.074, 0.092]	18.476	<0.0001
Kombewa	*An*. *funestus* density, *F*_2,77_ = 985.48, *P*<0.0001, adjusted *R* ^2^ = 0.96			
	A	6.507 [6.247, 6.768]	49.772	<0.0001
	B	−0.201 [−0.212, −0.189]	−35.341	<0.0001
	C	0.159 [0.148, 0.169]	29.370	<0.0001
Iguhu	*An*. *gambiae* density, *F*_2,95_ = 213.75, *P*<0.0001, adjusted *R* ^2^ = 0.81			
	A	5.239 [4.769, 5.709]	22.137	<0.0001
	B	−0.123 [−0.14, −0.105]	−13.92	<0.0001
	C	0.075 [0.061, 0.089]	10.496	<0.0001

Time series analysis revealed significant time-varying trend only in *An*. *gambiae* and *An*. *funestus* populations in Kombewa, and in *An*. *gambiae* population in Iguhu. Results of goodness-of-fit showed that model (EQ 2) fits all vector density data well with a prior setup of *D* = 0 (EQ 2) (Fig. 3 and Table 1). The resurgence of *An. gambiae* started from early 2008 in Kombewa and from early 2009 in Iguhu.

### ITNs coverage and efficacy

**Figure 5:**
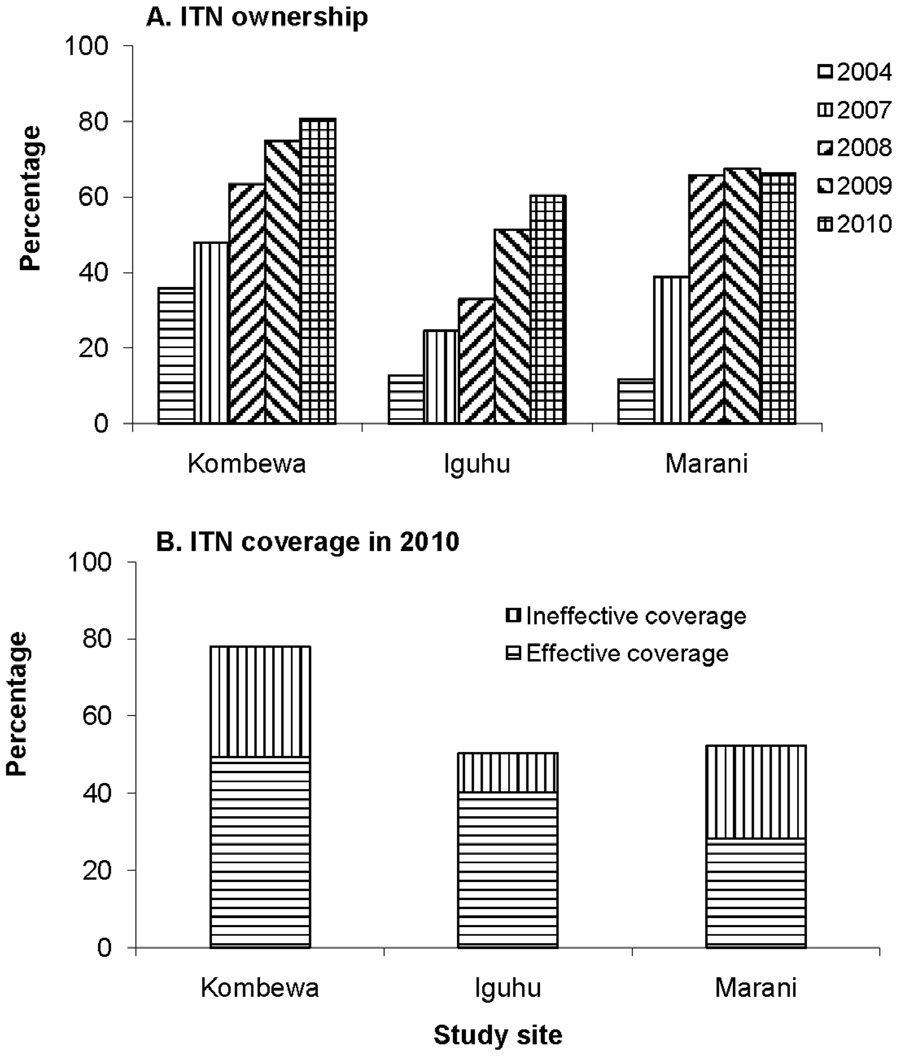
A) Increase in bed net ownership, presented as the percentage of households owning at least one net, from 2004 to 2010 (top); B) percentage of bed net coverage among the population in 2010 (bottom). Effective bed net coverage is defined as the proportion of the population who slept under LLINs in good condition, or slept under regular ITNs in good condition and that were routinely insecticide-retreated. Ineffective population coverage by bed net is defined as the proportion of the population who slept under torn nets, or slept under regular ITNs but not routinely insecticide-retreated.

Bed net ownership increased dramatically in Marani from 11.8% in 2004 to 65.8% in 2008 (χ^2^ = 98.9, df  = 1, P<0.0001), but it has remained almost unchanged since then (Fig. 5). In Iguhu, bed net ownership increased gradually from 12.8% in 2004 to 24.6% in 2007 and 60.3% in 2010 (χ^2^ = 55.5, df  = 1, P<0.0001). In Kombewa, bed net ownership was relatively high (35.9%) in 2004 and increased to 80.7% in 2010 (χ^2^ = 62.3, df  = 1, P<0.0001). Population bed net coverage, i.e., the population observed using the bed nets, had reached about 50.3% and 52.3% in Iguhu and Marani, respectively, and 77.9% in Kombewa in 2010. Even though bed net ownership and population bed net coverage were high, a substantial proportion of bed nets was torn or not-retreated ITNs. For example, we found that 20.1% (82/407), 36.6% (106/290), and 46.0% (88/191) of the bed nets being used during 2009 survey in Iguhu, Kombewa and Marani, respectively, were torn (with holes) or they were ITNs in good condition but were not retreated with insecticide. Furthermore, the 2010 survey found that 4.7% and 15.5% of the bed nets in Iguhu and Kombewa, respectively, were not used at all. Therefore, the functional effective bed net coverage in the population was estimated 40.3%, 49.4% and 28.2% in 2010 in Iguhu, Kombewa, and Marani, respectively (Fig. 5), which was much smaller than bed net ownership rate.

The result of contact bioassays of insecticidal test, indicated by mortality rate after 24 hours post exposure in the standard WHO paper cup test, showed that the overall efficacy LLINs varied among the study sites with 92.6% (ranged from 51–100%) and 81.1% (ranged from 24–100%) killing efficacy in Iguhu and Kombewa, respectively. Although older LLINs showed slightly lower efficacy than newer LLINs, ANOVA tests revealed no significant differences in efficacy across the age (in year) of LLINs in both Iguhu (efficacy ranged from 87.9–95.6%, *F*_4,22_ = 0.28, *P* = 0.89) and Kombewa (efficacy ranged from 70.7–93.4%, *F*_3,16_ = 0.47, *P* = 0.71). Due to the small number of conventional ITNs tested, the comparison between efficacies of different ages of nets was not performed.

In Iguhu, 14% of LLINs tested showed a mosquito-killing efficacy below 80% - the effective threshold set by WHOPES (World Health Organization Pesticide Evaluation Scheme). In Kombewa, approximately one quarter of the sampled bed nets caused 100% mosquito mortality and 75% of the tested LLINs showed a mortality rate above 80%, whereas 20% of the tested LLINs exhibited <50% killing efficacy, the minimum efficacy threshold set by WHOPES.

### Variation in climatic conditions

**Figure 6:**
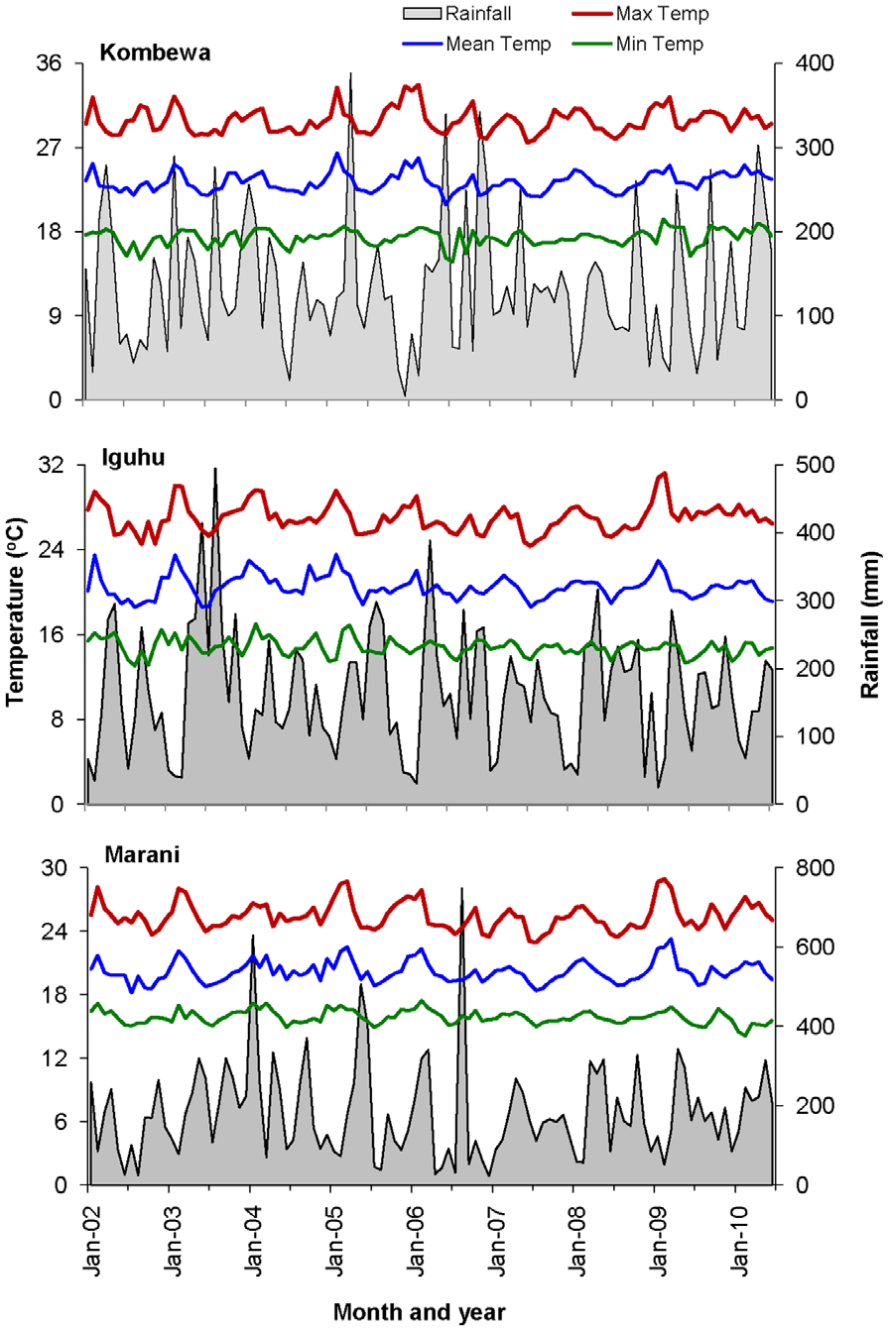
Variations in monthly maximum temperature, minimum temperature, mean temperature and monthly rainfalls in Kombewa (top), Iguhu (middle) and Marani (bottom) from 2002 to 2010.

Although there were variations in monthly cumulative precipitation and monthly mean maximum and minimum temperatures over the 9 years, the changing patterns in climatic parameters were not associated with changes in malaria parasite prevalence and vector abundances (Fig. 6), because no significant time-lagged correlation between parasite prevalence or vector abundance and climatic variables was found in any of the study sites.

## Discussion

Encouraged by the progress made in the last decade [1], [4], the malaria intervention paradigm has shifted from malaria control to malaria elimination in many parts of the world [7], [8]. Indeed, malaria eradication was set as an ultimate goal by WHO and the Roll-Back Malaria Partnership [39]. After a lapse of 40 years, malaria eradication is back on the global health agenda. Clinical malaria incidence has indeed declined by >50% in a number of malaria endemic countries in the last two decades [1], supporting the notion the current malaria intervention strategies, chiefly ITN, rapid diagnostic tests (RDT) and ACT treatment, are generally effective in combating malaria at least for the short term. However, the progress made in malaria control is not spatially congruent; the recent resurgence in clinical malaria has been reported from a few places in Kenya [18].

Malaria prevalence has exhibited a large change over the nine year study period in the three sentinel sites in western Kenya, as indicated by the initial decline of vector abundances and malaria prevalence resurgence since 2007. Without the long-term longitudinal monitoring, it is impossible to detect these changes, especially the rebounding of parasite prevalence and vector density. For example, point data did not show a significant difference in parasite prevalence in Kombewa between 2004 (53.9%) and 2009 (51.7%), leaving one to wonder if the bed net intervention policy in 2006 had any impact on malaria transmission at all. The reasons for the changing pattern of malaria epidemiology are most likely multi-factorial. The decreasing trend in *P. falciparum* prevalence was associated with a decline in vector abundance after nationwide implementation of free ITN/LLINs and increased use of ACT treatment for uncomplicated malaria.

Although large numbers of insecticide treated bed nets is being distributed in malaria endemic sub-Saharan Africa, population bed net coverage is still low and did not meet the targeted goal of 80% coverage at our study sites [40]. There are also concerns about the efficacy of the nets and long-term durability of insecticide-based ITN or IRS strategy. Evidence for the preventative effectiveness of bed nets in reducing vector densities and parasite prevalence is mixed and controversial. We observed substantial changes in vector abundance and malaria prevalence in all study sites in the first two years of ITN scaling-up and ACT use. However, three years later malaria vector abundance rebounded to a level similar to pre-scaling up of ITNs in Kombewa. Furthermore, we have observed a high proportion of *An*. *gambiae* s.s. relative to *An*. *arabiensis* in Kombewa and Iguhu in 2010, which was distinct to the sites in the same area in early years in western Kenya [36], [37]. This suggests that the efficacy of the bed nets is declining and/or malaria vectors are developing resistance to pyrethroid insecticides used in ITN impregnation only after a few years of use.

The gradual resurgence of parasite prevalence since 2007 may be explained by several factors. First, good conditions and proper use of bed nets are crucial for preventing parasite infections; the high percentage of torn bed nets and the erratic use of nets may have contributed to the resurgence observed, similar to previous findings [41]–[43]. Second, ITNs/LLINs have a time limited window of optimum efficacy after which sub-optimal performance sets in [42]. Third, the development of pyrethroid resistance in *Anopheles* mosquitoes might compromise the long-term effectiveness of ITNs and LLINs in killing mosquitoes. The pyrethroid resistant alleles, *kdr* alleles, have not been detected in either *An*. *gambiae* s.s. or *An*. *arabiensis* populations in the study areas in 2005 [44], but the preliminary results of an on-going study confirms that field caught *An*. *gambiae* s.s. in Kombewa and Iguhu are resistant to both permethrin and deltamethrin (Afrane, unpublished data). A change in the vector biting behavior was observed in the study areas, with mosquitoes tending to bite earlier, before bed time (Githeko, unpublished data) [45], [46]. The emergence of this new vector behavioural phenotypes is a less-recognized phenomenon than insecticide resistance, but it has the potential to similarly diminish the effectiveness of current interventions. It is more likely due to a combination of phenotypic (behaviour change) and genotypic (insecticide resistance) changes that have diminished the efficacy of bed nets [47], [48]. The variations in climate, especially the changes in daily maximum and minimum temperature in western Kenya, were unlikely to play a major role in the dynamic shift of vector populations or parasite prevalence. Additionally, parasite resistance to antimalarial drugs is a critical issue that needs close observation.

Malaria control in Africa has focused on young children (<5 years age group) and pregnant women over the last decade, but as transmission intensity declines, school aged children will become an important clinical risk group. Brooker et al [49] reviewed the historical experience and current rationale for the use of schools and school children as a complementary, inexpensive framework for evaluating malaria control in Africa. In this study, parasitological surveys focused mainly on school children because of several reasons. Previous studies found that the risks and severity of clinical outcomes, following exposure to *P*. *falciparum,* increase among older children as transmission intensity declines because ITN coverage was significantly lower in school-aged children compare to both pre-school children and adults [50], [51]. Thus, school children are a sensitive age group for malaria prevalence determination and for evaluating the impacts of malaria intervention measures. Additionally, there are other important epidemiological reasons for sampling children who attend school. Historically, malaria endemicity was defined on the basis of *Pf* PR (*Plasmodium falciparum* parasite rate) among children aged 2–10 years. Our study provides information on parasite prevalence in slightly older children. Furthermore, the technical capacity to conduct school malaria surveys already exists in most sub-Saharan Africa countries [49].

Current integrated malaria management, including ITNs, IRS, ACT and RDTs, has and will continue to have a substantial impact on malaria morbidity and mortality in Kenya. There was a clear association between the steep decrease in vector abundance and the mass distribution of ITNs. However, the impact was short lived, and it seems that there are major gaps in ITN ownership and actual use. World Health Organization has issued the guidelines on proper use and sustaining high coverage and replacement of old LLINs (3–5 y) in 2005 [26], but it seems that the guideline has not yet been followed and implemented in the study areas. In addition, since the progress was achieved through unprecedented amount of donor funding [20], [52], without additional and sustained external funds the sustainability of the success achieved is questionable [49]–[51]. Furthermore, since the principal determinants of malaria burden in Africa are linked to ecological and societal changes, climate, politics, and economics, these factors and their interactions remain complicated and the volatility of some political or economic changes [53]–[57] may negatively affect the systematic implementation of large-scale malaria control operation [11], [58]–[60]. These further highlight the challenges in sustained malaria control in Africa and other regions. To evaluate the impacts of the current intensive malaria control measures or to determine the changing malaria epidemiology, a strong surveillance system for malaria vectors, parasite prevalence and clinical cases must be established in the sub-Saharan Africa.
